# Milk microfiltration process dataset annotated from a collection of scientific papers

**DOI:** 10.1016/j.dib.2021.107063

**Published:** 2021-04-17

**Authors:** Patrice Buche, Stéphane Dervaux, Nadine Leconte, Maellis Belna, Manon Granger-Delacroix, Fabienne Garnier-Lambrouin, Gustavo Gregory, Lucille Barrois, Geneviève Gesan-Guiziou

**Affiliations:** a IATE, University of Montpellier, INRAE, Institut Agro, Montpellier, France; b STLO, INRAE, Institut Agro, 35000 Rennes, France; c Boccard, Research and Development, F-35360 Montauban-de-Bretagne, France; d Department of Research & Innovation, Sodiaal International, 75014 Paris, France; e MIA, AgroParisTech, INRAE, Paris, France

**Keywords:** Milk, Microfiltration, Process, Performances, Operating conditions, Composition characterization, Protein fractionation, Ontology

## Abstract

Milk microfiltration process plays a key role in the dairy industry. Crossflow microfiltration of skimmed milk using a membrane with 0.1 µm mean pore size is widely used to fractionate the two main groups of dairy proteins: casein micelles (~150 nm) and serum proteins (~2-15 nm). Retentate, containing mainly casein micelles, is generally used to enrich vat milk for cheese making. Permeate, containing serum proteins, lactose and minerals, is usually ultrafiltered in order to produce protein-rich concentrate with a high nutritional value dedicated to specific populations such as infants and seniors. The great interest in these protein fractions explains the increasing number of microfiltration equipments in the dairy industry. This data article contains data associated with milk microfiltration process experiments and properties of the resulting dairy fractions annotated from a collection of scientific documents. These data are stored in INRAE public repository (see Data accessibility in the Specification Table for direct links to data). They have been structured using MILK MICROFILTRATION ontology and are replicated in @Web data warehouse providing additional querying tools (https://www6.inrae.fr/cati-icat-atweb/).

## Specifications Table

**Table:** 

Subject	*Milk microfiltration*
Specific subject area	A dataset of milk microfiltration process experiments and properties of the resulting dairy fractions were annotated from a collection of scientific documents
Type of data	Table
How data were acquired	A collection of 190 experiments annotated from 56 scientific documents from 1987 (year of the first publication on milk microfiltration directed to the separation of casein micelles from serum proteins) to 2019 extracted from Web Of Science (WOS) and other sources
Data format	Raw and analyzed
Parameters for data collection	Experiments have been classified in 4 process types namely HT-MF, HT-MF-DF, RB-MF and RB-MF-DF depending on the sequence of unit operations: HT-MF corresponds to process experiments in which a heat treatment (HT) precedes a milk microfiltration (MF) unit operation; HT-MF-DF corresponds to process experiments in which a heat treatment precedes a milk microfiltration which precedes a diafiltration unit operation (DF); RB-MF corresponds to process experiments in which a removal of bacteria by microfiltration (RB) precedes a milk microfiltration unit operation; RB-MF-DF corresponds to process experiments in which a removal of bacteria by microfiltration precedes a milk microfiltration which precedes a diafiltration unit operation.
Description of data collection	For each of the 4 process types, data from the milk microfiltration process experiments were collected in 5 tables (files). Table Process Description: Processing parameters (sequence of unit operations associated with process experiments, machine-operating conditions, measured parameters …). Table Controlled Parameter Evolution: measured parameters at different sampling times during the milk microfiltration operation for each process experiment registered in Table Process Description. Table Sample Identification: List of samples extracted at the end of unit operations of a given process experiment registered in Table Process Description. Table Sample Characterization: Characterization data of all samples registered in Table Sample Identification (pH, turbidity, lightness, dry matter …). Table Reliability Assessment: a collection of 8 metadata used for data reliability assessment associated with each annotated document.
Data source location	*INRAE, FR-75000, Paris, France* https://doi.org/10.14758/9T8G-WJ20 *The list of 56 annotated* documents *is given in Appendix 1.*
Data accessibility	Data are accessible in a public repository Repository name: INRAE dataverse (https://data.inrae.fr/) Data identification number: 10.15454/0ADYFY corresponding to HT-MF process experiments, 10.15454/EFC7VP corresponding to RB-MF process experiments, 10.15454/7NOR3F corresponding to HT-MF-DF process experiments, 10.15454/A4KXE7 corresponding to RB-MF-DF process experiments, 10.15454/G3YP4O corresponding to the list of unannotated documents, 10.15454/5MQMKG corresponding to the MILK MICROFILTRATION ontology. Direct URL to data: https://doi.org/10.15454/0ADYFY (v6.0) corresponding to HT-MF process experiments, https://doi.org/10.15454/EFC7VP (v4.0) corresponding to RB-MF process experiments, https://doi.org/10.15454/7NOR3F (v3.0) corresponding to HT-MF-DF process experiments, https://doi.org/10.15454/A4KXE7 (v3.0) corresponding to RB-MF-DF process experiments, https://doi.org/10.15454/G3YP4O (v5.0) corresponding to the list of unannotated documents, https://doi.org/10.15454/5MQMKG (v1.3) corresponding to the MILK MICROFILTRATION ontology.

## Value of the Data

• A unique set of annotation data about operating conditions and performances of skimmed milk microfiltration applied to the fractionation of dairy proteins proposed in the scientific literature.
• These data can be used to analyze and compare skimmed milk microfiltration performance in a large range of operating conditions and membrane configurations and systems.
• These data could serve as benchmark for researchers and industrials, both dairy users and equipment providers, coping with skimmed milk microfiltration and dairy protein fractionation.

## 1 Data Description

Crossflow microfiltration process of milk using a 0.1 µm mean pore size membrane plays a key role in the dairy industry. This process is widely used to fractionate the two main groups of dairy proteins. Casein micelles (~150 nm), concentrated in the retentate, are generally used to enrich vat milk for cheese making and serum proteins (~2-15 nm), contained in the permeate, are usually ultrafiltered in order to produce protein-rich ingredients with a high nutritional value.

The principle of crossflow filtration is to separate components from a fluid (milk in our case) circulating tangentially to a membrane by applying a driving force between the two sides of the membrane. The Volume Reduction Ratio, VRF is the ratio of feed flow rate to retentate extraction flow rate and is used in industry to adjust the retentate extraction flow rate to obtain a targeted concentration of the component in the retentate fraction. TMP is the pressure that forces the fluid to pass through the membrane and is defined as the difference of pressure between the retentate and permeate sides. JP characterizes the mass flow passing through the membrane; Shear stress and crossflow velocity both characterise the erosion at the membrane surface. With crossflow filtration, the tangential motion of the fluid across the membrane causes retained particles at the membrane surface to be rubbed off. This article contains data associated with milk microfiltration process experiments and properties of the resulting dairy fractions annotated from a collection of scientific documents.

**Fig. 1:**
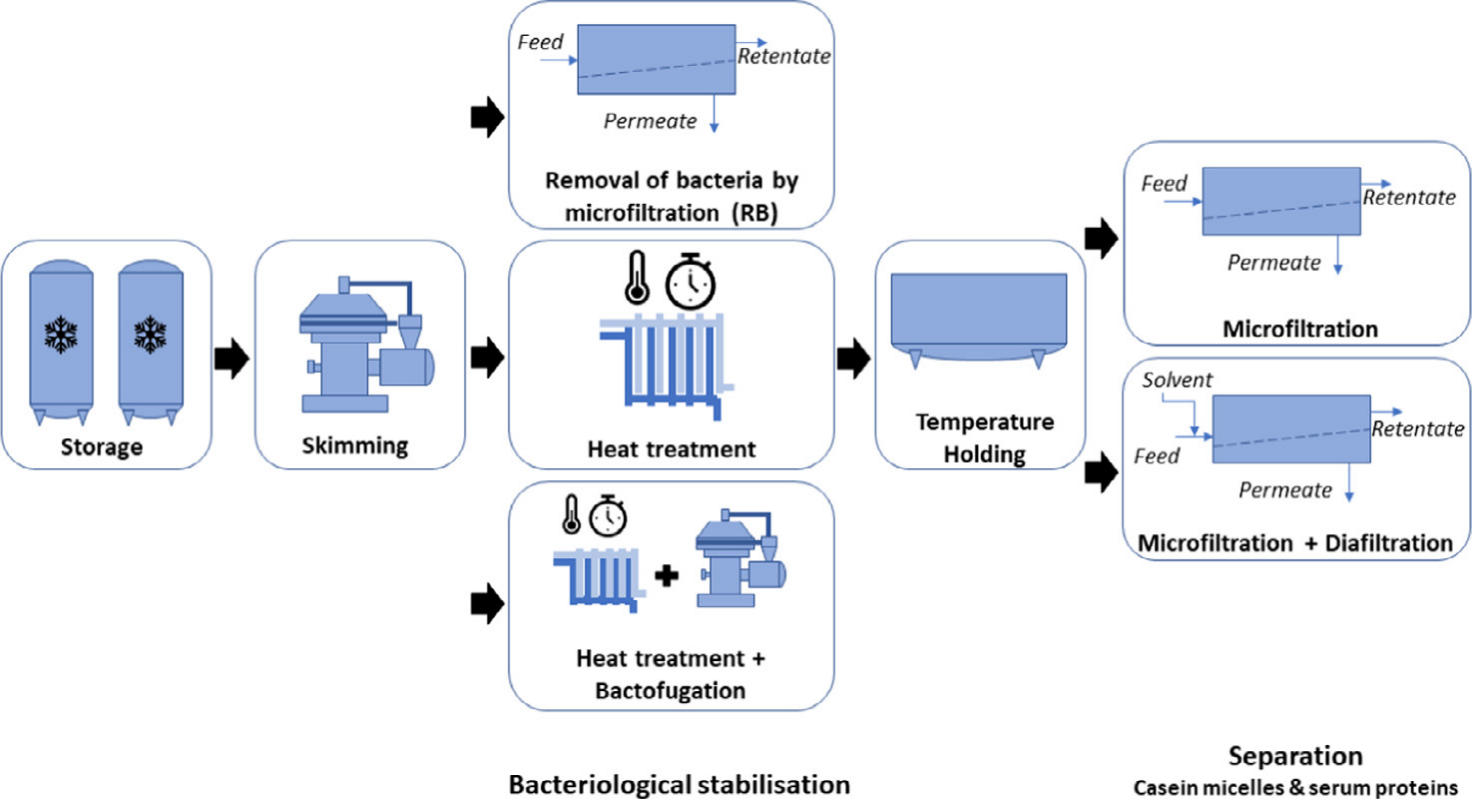
Possible sequences of unit operation associated with the 4 considered process types.

Data are stored in five datasets (see Data accessibility in the Specification Table above). The four first ones contain process experiments classified in four process types namely HT-MF, HT-MF-DF, RB-MF and RB-MF-DF (see Fig. 1). Each process type is characterized by a given sequence of unit operations: HT-MF corresponds to process experiments in which a heat treatment precedes a milk microfiltration unit operation; HT-MF-DF corresponds to process experiments in which a heat treatment precedes a milk microfiltration which precedes a diafiltration unit operation; RB-MF corresponds to process experiments in which a removal of bacteria by microfiltration precedes a milk microfiltration unit operation; RB-MF-DF corresponds to process experiments in which a removal of bacteria by microfiltration precedes a milk microfiltration which precedes a diafiltration unit operation.

The fifth dataset contains the list of documents that have not been annotated because they did not include the requested information. Documents have been annotated when it was possible to answer the following questions:

1. Which are the different unit operations of a given microfiltration process experiment?
2. Which are the measured parameter values associated with a given unit operation of the process experiment?
3. Which are the measured parameter value evolutions and associated sample characteristics during the microfiltration unit operation?
4. Which are the characteristic values associated with samples extracted at the end of unit operations of a given process experiment?

In each of the four datasets corresponding to a given process type, the data are gathered in five files. File Process Description XX^1^ provides a precise description of milk microfiltration process experiments associated with produced milk samples. File Sample Identification XX lists the samples extracted at the end of unit operations of a given itinerary registered in File Process Description XX. File Sample Characterisation XX contains the data associated with characteristics of all milk samples listed in File Sample Identification XX. The kinetics of measured parameter values and milk sample characteristics during the microfiltration unit operation are gathered in file Controlled Parameter Evolution XX. An additional file Reliability Assessment contains a set of 8 metadata values associated with each annotated article. Those metadata can be used for data reliability assessment [1], [3].

In File Process Description XX, a process experiment is described by a set of unit operations and associated with a unique numerical identifier (column *Experience number*). Each unit operation of a given process experiment is described on a separate line and associated with a unique numerical identifier (columns *Experience number* + *Process step number*). Numerical value associated with *Process step number* column indicates temporal order (e,g step 1 is before step 2). The following information are reported if available in the article: processing operation type (Treatment) and complementary information (Treatment_ORIGINALVALUE); type of milk input of the operation (Input product) and complementary information (Input product_ORIGINALVALUE); treatment duration; temperature; rotation speed; membrane reference; manufacturer; membrane material; membrane configuration; membrane system; working mode; molecular weight cut-off; pore size; total membrane area; number of membrane elements; number of channel per membrane element; membrane length; spiral spacer size; flat sheet liquid film thickness; tubular channel diameter; diafiltration mode; solvent nature.

File Sample Identification XX contains the list of samples extracted at the end of unit operations of a given process experiment registered in File Process Description XX. Each sample is associated with a unique identifier (columns *Experience number* + *Sample number*). Column Product characterizes the type of produced sample (output permeate, output retentate …).

Characteristics associated with samples are stored in file Sample Characterisation XX. Each line contains the set of characteristics associated with a given sample. Values associated with following characteristics are stored if available in the document: initial product state; sampling time; pH; turbidity; lightness; dry matter; ash; total calcium content; phosphorus content; fat content; lactose; total protein; npn; ncn; true protein; cn-Kjeldahl; sp-Kjeldahl;cn%tp; cn-hplc; Serum protein-HPLC; serum protein-SDS-Page; beta-Lactoglobulin transmission; alpha-Lactalbumin transmission; Immunoglobulin; lactoferrin; bsa; casein-β; density; viscosity; total microbial count; cheesemaking process technological level; rennet clotting time; time to 20 mm firmness; gel firmness; cheese yield.

Measured parameters at different sampling times during the milk microfiltration operation for each itinerary registered in File Process Description are stored in file Controlled Parameter Evolution XX. Values associated with following characteristics are stored if available in the document: sampling time; process phase; volume reduction factor, VRF; concentration factor, CF; temperature; transmembrane pressure, TMP; Permeation flux, Jp; feed flow, Q; crossflow velocity; retentate pressure drop; shear stress at the membrane surface; transmission of serum proteins, SP transmission; removal of serum proteins, SP removal; β-Lactoglobulin transmission; α-Lactalbumin transmission.

File reliability assessment XX contains a list of vectors composed of 8 metadata used for data reliability assessment, each vector being associated with an annotated document. The list of metadata is: source type; assay type; number of repetitions for microfiltration assays; operating mode; number of automatic parameter controls during MF; initial product state; protein analysis method; number of repetitions for compositional analysis.

These data are stored in INRAE dataverse and replicated in @Web data warehouse [2] (https://www6.inra.fr/cati-icat-atweb/) in which the data structuration and vocabulary standardization are controlled by MILK MICROFILTRATION ontology. MILK MICROFILTRATION ontology is available in INRAE dataverse (https://doi.org/10.15454/5MQMKG, version 1.3) and Agroportal (http://agroportal.lirmm.fr/ontologies/MICROFILTRATION, version 2.1). MILK MICROFILTRATION ontology defines a standardized vocabulary used to annotate and structure data retrieved from the documents in a homogeneous way. A short description of Milk Microfiltration is available on @Web companion web site (https://www6.inrae.fr/cati-icat-atweb/Ontologies/Microfiltration) and additional explanations about the ontological model used may be found in [1]. Two ways to browse the vocabulary are provided: (1) using @Web application (see a short description of Milk Microfiltration and explanation how to browse it on @Web companion web site - https://www6.inrae.fr/cati-icat-atweb/Ontologies/Microfiltration; (2) using AgroPortal browser (http://agroportal.lirmm.fr/ontologies/MICROFILTRATION, tab Classes).

Tutorials associated with atWeb querying module are available at https://www6.inrae.fr/cati-icat-atweb/Tutorials/Querying-thumbnail.

## 2 Experimental Design, Materials, and Methods

The dataset annotated with the Microfiltration process ontology is composed of 56 scientific documents (articles, thesis, scientific reports) upon a total of 213 documents. The 213 documents were selected from Web of Science and other sources with the keywords “Microfiltration” + “Milk” and filtered by experts to keep only documents in the field of conventional milk microfiltration directed to the separation of casein micelles from serum proteins. Documents have been discarded from the initial set of 213 documents. We identified two main kinds of discarded documents:

i) 96 documents were out of the scope of conventional milk microfiltration. For instance they use dynamic filtration, ultrasound or gas injection, or specifically study the analysis of membrane fouling without reporting microfiltration operating conditions;
ii) 116 other documents did not provide enough data to answer the four questions listed in the data section of the paper. Indeed some initially listed documents used microfiltration process to study the impact of this process on cheese manufacture and did not provide detailed operating parameters of microfiltration. Others, mainly patents, reviews, technical papers or scientific papers dealing with hydraulic performance modelling, did not include requested information on product characteristics.

The list of documents, which have been discarded, is given in the dataset https://doi.org/10.15454/G3YP4O.

The list of the 56 annotated documents is given in Appendix 1.

The annotations realized in the set of 56 relevant documents include:

• 190 process experiments,
• 1572 microfiltration controlled parameters evolution at different sampling times,
• 731 sample characterizations.

The annotation process of the 56 scientific documents has been contacted in two steps. In the first one, a double-blind annotation has been done on a subset of five documents answering to the four questions listed previously. The annotators have fulfilled the five files described previously for each article. The comparison of double-blind annotations has permitted to identify and solve annotation ambiguities. The resulting conclusions have been written as annotation guidelines. The remaining documents have been annotated using these guidelines to homogenize annotations. The annotation guidelines can be found in each of the four datasets associated with the four process types.

For each document, annotators have registered eight metadata, which can be used for reliability assessment following the method proposed in [3]. Metadata (resp.-associated values) are listed in the following table in columns Group (resp. Meta-information values) (Table 1).

**Table 1:** List of metadata used for data reliability assessment.

Type of group	Group	Meta-information values
meta-information on the data source itself	MF-Source Type	Book Proceeding Journal article superior IF quarter (in at least one area) Journal article second IF quarter (in at least one area) Journal article third IF quarter (in at least one area) Journal article inferior IF quarter (in at least one area) Encyclopedia article Patent Report Thesis Science popularization article

meta-information related to means used to produce data	MF-Assay type	Parametric As a function of time (>2h) As a function of time (<2h) Other

	MF-Operating mode	Continuous (ceramic UTP/GP) Batch (ceramic UTP/GP) Continuous (ceramic Other) Batch (ceramic Other) Continuous or batch (Polymeric) Non specified

	MF-Number of automatic parameter controls during MF (PTM, J, v, VRF, T, σ)	4 or more 3 2 1 0 Non specified

MF-Initial product state	Liquid milk Powder milk (low heat) Powder milk Others

MF-Protein analysis method	HPLC SDS-Page Kjeldahl Other None

meta-information related to statistical procedures	MF-Number of repetitions – Microfiltration assays	2 or more 1
	MF-Number of repetitions – Compositional analysis	3 or more 2 1

## Ethics Statement

This work neither involves human subject nor animal experiments.

## CRediT Author Statement

**Patrice Buche:** Methodology, Software, Data curation, Writing - Original Draft - Review & Editing; **Stéphane Dervaux:** Software, Data curation; **Nadine Leconte**: Investigation, Data curation, Validation; **Maellis Belna**: Conceptualization, Methodology, Investigation, Validation, Writing - Original Draft; **Manon Granger-Delacroix** Investigation, Data curation, Validation; **Fabienne Garnier-Lambrouin** Methodology, Validation; **Gustavo Gregory** Conceptualization, Methodology, Investigation, Validation, Writing; **Lucille Barrois** Investigation, Data curation, Validation; **Geneviève Gesan-Guiziou** Supervision, Conceptualization, Methodology, Investigation, Validation, Writing.

## Declaration of Competing Interest

The authors declare that they have no known competing financial interests or personal relationships, which have, or could be perceived to have, influenced the work reported in this article.

## Appendix Supplementary materials

Image, application 1

## Footnotes

1 XX being HT-MF or HT-MF-DF or RB-MF or RB-MF-DF.
