# Supplementary material for: Milk microfiltration process dataset annotated from a collection of scientific papers
Source: Data Brief. 2021 Apr 17;36:107063. doi: 10.1016/j.dib.2021.107063 (PMC8131563; doi:10.1016/j.dib.2021.107063)
Supplement: Supplementary file 1 [file mmc1.docx]

**Appendix 1 List of annotated documents**

Each document is associated with one or several process types namely HT-MF, HT-MF-DF, RB-MF and RB-MF-DF (see definition of the categories in section Data). By example, reference 1 is associated with HT-MF-DF, which means that process experiments annotated in this document belong to HT-MF-DF process type. Reference 3 is associated with HT-MF and HT-MF-DF, which means that process experiments annotated in this document belong to HT-MF or HT-MF-DF process types.

1. Aaltonen, T and Huumonen, I.;Ripening of cheese made from full concentrated milk retentate with and without peptidase addition;;International Dairy Journal;13;12;P 945-951.;;2003;http://doi.org/10.1016/S0958-6946(03)00143-2;HT-MF-DF
2. Adams, M. C. and Barbano, D. M.;Effect of ceramic membrane channel diameter on limiting retentate protein concentration during skim milk microfiltration;;Journal of Dairy Science;88;5;P1891-1900.;;2005;http://doi.org/10.3168/jds.S0022-0302(05)72865-4;HT-MF-DF
3. Adams, M. C. and Barbano, D. M.;Serum protein removal from skim milk with a 3-stage, 3x ceramic Isoflux membrane process at 50°C;;Journal of Dairy Science;99;7;P 5230-5243.;;2016;http://doi.org/10.3168/jds.2016-10914;HT-MF, HT-MF-DF
4. Adams, M. C., Hurt, E. E., Barbano, D. M.;Effect of ceramic membrane channel geometry and uniform transmembrane pressure on limiting flux and serum protein removal during skim milk microfiltration.;;Journal of Membrane Science;158;;P 211-222.;;1999;http://doi.org/10.1016/S0376-7388(99)00017-4;HT-MF
5. Adams, M. C., Hurt, E. E., Barbano, D. M.;Effect of soluble calcium and lactose on limiting flux and serum protein removal during skim milk microfiltration;;Proceedings ICOM Heidelberg, Germany;;;;;1993;;HT-MF
6. Adams, M. C., Zulewska, J., Barbano, D. M.;Effect of annatto addition and bleaching treatments on ultrafiltration flux during production of 80% whey protein concentrate and 80% serum protein concentrate;;Lait;74;;P 47-63;;1994; https://doi.org/10.1051%2Flait%3A199415;RB-MF
7. Amelia, I. and Barbano, D. M.;Production of an 18% protein liquid micellar casein concentrate with a long refrigerated shelf life;;Lait ;74;;P 375-388;;1994;;RB-MF-DF
8. Ardisson-Korat, A. V., Rizvi, S. S.H.;Vatless Manufacturing of Low-Moisture Part-Skim Mozzarella Cheese from Highly Concentrated Skim Milk Microfiltration Retentates;;Journal of Dairy Science;82;10;P 2063-2069.;;1999;http://doi.org/10.3168/jds.S0022-0302(99)75447-0;HT-MF
9. Astudillo-Castro, C. L.;Limiting Flux and Critical Transmembrane Pressure Determination Using an Exponential Model: The Effect of Concentration Factor, Temperature, and Cross-Flow Velocity during Casein Micelle Concentration by Microfiltration;;Journal of Dairy Science;96;4;P 2035-2047.;;2013;http://doi.org/10.3168/jds.2012-6009;HT-MF
10. Attia, H.;Ultrafiltration du lait, de laits acidifiés et de caillés lactiques sur membrane minérale;;Journal of Dairy Science;99;;P 167-182.;;2016;http://doi.org/10.3168/jds.2015-9897;HT-MF
11. Attia, H., Bennasar, M., De la Fuente, B. T.;Study of the fouling of inorganic membranes by acidified milks using scanning electron microscopy and electrophoresis. I. Membrane with pore diameter 0·2 μm;;Journal of Dairy Science;98;11;P 7527-7543.;;2015;http://doi.org/10.3168/jds.2015-9753;HT-MF
12. Beckman, S. L. and D. M. Barbano;Effect of microfiltration concentration factor on serum protein removal from skim milk using spiral-wound polymeric membranes;;Journal of Dairy Science;96;10;P 6199-6212.;;2013;http://doi.org/10.3168/jds.2013-6655;HT-MF
13. Beckman, S. L., Zulewska, J., Newbold, M., Barbano, D. M.;Production efficiency of micellar casein concentrate using polymeric spiral-wound microfiltration membranes;;Journal of Dairy Science;98;11;P 7483-7497.;;2015;http://doi.org/10.3168/jds.2015-9474;HT-MF
14. Beliciu, C. M., Sauer, A., Moraru, C. I.;The effect of commercial sterilization regimens on micellar casein concentrates;;Journal of Dairy Science;92;4;P 1361-1377.;;2009;http://doi.org/10.3168/jds.2008-1757;HT-MF
15. Brandsma, R. L., Rizvi, S. S. H.;Depletion of Whey Proteins and Calcium by Microfiltration of Acidified Skim Milk Prior to Cheese Making;;Journal of Membrane Science;189;;P 69-82;;2001;https://doi.org/10.1016%2Fs0376-7388(01)00396-9;HT-MF
16. Brandsma, R. L., Rizvi, S. S. H.;Manufacture of Mozzarella cheese from highly-concentrated skim milk microfiltration retentate depleted of whey proteins;;Lait;80;1;P 5-14;;2000;http://doi.org/10.1051/lait:2000100;HT-MF
17. Crowley. S. V. , Caldeo. V. , McCarthy. N. A. , Fenelon. M. A , Kelly. A. K. , O’Mahony J.;Processing and protein-fractionation characteristics of different polymeric membranes during filtration of skim milk at refrigeration temperatures;;International Dairy Journal;74;1;P 12-20.;;2017;http://doi.org/10.1016/j.idairyj.2016.11.018;HT-MF
18. Dong, J. Y., Chen, L. J., Maubois, J. L., Ma, Y.;Influence of medium-concentration factor microfiltration treatment on the characteristics of low-moisture Mozzarella cheese;;Proceedings 25th International Dairy Congress;;;;;1998;;RB-MF
19. Fauquant, J., Maubois, J.-L., Pierre, A.;Microfiltration of milk using a mineral membrane;;Journal of Membrane Science;352;;P 107-115.;;2010;http://doi.org/10.1016/j.memsci.2010.02.006;HT-MF
20. Gésan-Guiziou, G., Boyaval, E., Daufin, G.;Critical stability conditions in crossflow microfiltration of skimmed milk: transition to irreversible deposition;;Journal of Dairy Science;96;;P 1-13;;2013;https://doi.org/10.3168/jds.2012-6032;HT-MF
21. Gésan-Guiziou, G., Daufin, G., Boyaval, E., Le Berre, O.;Wall shear stress: effective parameter for the characterisation of the cross-flow transport in turbulent regime during skimmed milk microfiltration;;Dairy Science & Technology;89;2;P 139-154.;;2009;http://doi.org/10.1051/dst/2009002;RB-MF
22. Grangeon, A., Lescoche, P.;Flat ceramic membranes for the treatment of dairy products: comparison with tubular ceramic membranes;;Journal of Membrane Science;369;;P 404-413;;2011;http://doi.org/10.1016/j.memsci.2010.12.026;HT-MF
23. Hartinger, M. , Heidebrecth, H. J. , schiffer, S. , Dumpler, J. , Kulozik, U.;Milk protein fractionation by means of spiral-wound microfiltration membranes : effect of the pressure adjustment mode and temperature on flux and protein permeation;;Industrial & Engineering Chemistry Research;54;1;P 414-425.;;2015;http://doi.org/10.1021/ie5033292;HT-MF
24. Heino, A. T., Uusi-Rauva, J.O., Outinen, M.;Microfiltration of milk I: Cheese milk modification by micro-and ultrafiltration and the effect on Emmental cheese quality;;International Journal of Food Science and Technology;36;6;P 611-624.;;2001;http://doi.org/11.1046/j.1365-2621.2001.00507.x;HT-MF
25. Heino, A. T., Uusi-Rauva, J.O., Outinen, M.;Microfiltration of milk III: Impact of milk modification on milk coagulation kinetics;;Journal of Dairy Science;93;12;P 5588-5600;;2010;http://doi.org/10.3168/jds.2010-3169;HT-MF-DF
26. Heino, A. , Outinen, M. , Uusi-Rauva, J.;Removal of whey proteins from skimmed milk with polymeric microfiltration membranes;;Milchwissenschaft;63;3;P 279-283.;;2008;;HT-MF, HT-MF-DF
27. Horst, C. van der, Piersma, I.;Crossflow microfiltration of skim milk;;Milchwissenschaft;63;3;P 305-308.;;2008;;HT-MF, HT-MF-DF
28. Hurt, E. E. , Adams, M. C., Barbano, D. M.;Microfiltration: Effect of channel diameter on limiting flux and serum protein removal;;Milchwissenschaft;64;2;P 128-131.;;2009;;HT-MF, HT-MF-DF
29. Hurt, E. E. , Adams, M. C., Barbano, D. M.;Microfiltration: Effect of retentate protein concentration on limiting flux and serum protein removal with 4-mm-channel ceramic microfiltration membranes;;Technique Laitière & Marketing;1028;;P 21-23.;;1988;;HT-MF
30. Hurt, E. E., Adams, M. C., Barbano, D. M.;Microfiltration of skim milk and modified skim milk using a 0.1-µm ceramic uniform transmembrane pressure system at temperatures of 50, 55, 60, and 65°C;;Journal of Dairy Science;98;2;P 765-780.;;2015;http://doi.org/10.3168/jds.2014-8775;HT-MF
31. Hurt, E., Zulewska, J., Newbold, M., Barbano, D. M.;Micellar casein concentrate production with a 3X, 3-stage, uniform transmembrane pressure ceramic membrane process at 50°C;;Journal of Dairy Science;98;6;P 3599-3612.;;2015;http://doi.org/10.3168/jds.2014-9225;HT-MF
32. Jimenez-Lopez, A. J. E., Leconte, N., Dehainault, O., Geneste, C., Fromont, L., Gésan-Guiziou, G.;Role of milk constituents on critical conditions and deposit structure in skim milk microfiltration (0.1µm);;Journal of Dairy Science;98;4;P 2224-2234.;;2015;http://doi.org/10.3168/jds.2014-9032;HT-MF
33. Jimenez-Lopez, A., Leconte, N., Garnier-Lambrouin, F., Bouchoux, A., Rousseau, F., Gésan-Guiziou, G.;Ionic strength dependence of skimmed milk microfiltration: Relations between filtration performance, deposit layer characteristics and colloidal properties of casein micelles;;Foods;8;6;P 180;;2019;https://doi.org/10.3390/foods8060180;HT-MF
34. Jorgensen, C. E., Abrahamsen, R. K., Rukke, E.-O., Johansen, A.-G., Schüller, R. B., Skeie, S. B.;Optimization of protein fractionation by skim milk microfiltration: Choice of ceramic membrane pore size and filtration temperature;;International Dairy Journal;7;;P 237-242;;1997;https://doi.org/10.1016/S0958-6946(97)00009-5;HT-MF
35. Jorgensen, C. E., Abrahamsen, R. K., Rukke, E.-O., Johansen, A.-G., Skeie, S. B.;Fractionation by microfiltration: Effect of casein micelle size on composition and rheology of high protein, low fat set yoghurt;;Journal of Dairy Science;99;8;P 6164-6179.;;2016;http://doi.org/10.3168/jds.2016-11090;HT-MF
36. Jost, R., Brandsma, R., Rizvi, S.;Protein composition of micellar casein obtained by cross-flow micro-filtration of skimmed milk;;International Dairy Journal;18;3;P 236-246.;;2008;http://doi.org/10.1016/j.idairyj.2007.08.011;HT-MF
37. Kücükcetin, A., Yaygin, H., Hinrichs, J., Kulozik, U.;Adaptation of bovine milk towards mares’ milk composition by means of membrane technology for koumiss manufacture;;LWT- Food science and Technology;43;;P 647-654;;2010;https://doi.org/10.1016/j.lwt.2009.11.004;HT-MF
38. Kühnl, W., Piry, A., Kaufmann, V., Grein, T., Ripperger, S., Kulozik, U.;Impact of colloidal interactions on the flux in cross-flow microfiltration of milk at different pH values: A surface energy approach;;International Dairy Journal;48;;;;2015;https://doi.org/10.1016/j.idairyj.2015.01.005;HT-MF
39. Le Berre, O., Daufin, G.;Skimmilk crossflow microfiltration performance versus permeation flux to wall shear stress ratio;;Journal of Dairy Science;93;10;P 4506-4517;;2010;https://doi.org/10.3168/jds.2010-3261;HT-MF-DF
40. Marcelo, P. A., Rizvi, S. S. H.;Physicochemical properties of liquid virgin whey protein isolate;;Journal of Dairy Science;96;5;P 3340-3349.;;2013;http://doi.org/10.3168/jds.2012-6033;HT-MF-DF
41. Maubois, J.-L.;Fractionation of milk proteins;;International Dairy Journal;9;3-6;P 389-390.;;1999;https://doi.org/10.1016/S0958-6946(99)00101-6 ;RB-MF-DF
42. Nelson, B. K. and Barbano, D. M.;A Microfiltration Process to Maximize Removal of Serum Proteins from Skim Milk Before Cheese Making;;Milchwissenschaft;65;1;P 3- 6;;2010;;HT-MF
43. Nelson, B. K. and Barbano, D. M.;Yield and Aging of Cheddar Cheeses Manufactured from Milks with Different Milk Serum Protein Contents;;Milchwissenschaft-Milk Science International;52;4;P 187-192;;1997;;HT-MF
44. Outinen, M., Heino, A., Uusi-Rauva, J.;Microfiltration of milk II. Influence of the concentration factor on the composition of Emmental cheese milk and the ?-casein macropeptide content of the whey;;International Journal of Dairy Technology;66;2;P 214-219.;;2013;http://doi.org/10.1111/1471-0307.12039;HT-MF-DF
45. Outinen, M., Heino, A., Uusi-Rauva, J.;Pre-treatment methods of Edam cheese milk. Effect on the whey composition;;Separation and Purification Technology;61;1;P 33-43.;;2008;http://doi.org/10.1016/j.seppur.2007.09.023;HT-MF
46. Samuelsson, G. , Dejmek, P. , Tragardh, G. , Paulsson, M.;Minimizing whey protein retention in cross-flow microfiltration of skim milk;;Journal of Dairy Science;96;4;P 2020-2034.;;2013;http://doi.org/10.3168/jds.2012-6007;HT-MF-DF
47. Samuelsson, G. , Dejmek, P. , Tragardh, G. , Paulsson, M.;Rennet coagulation of heat treated retentate from crossflow microfiltration of skim milk;;Journal of Dairy Science;101;;P 10860-10865;;2018;10.3168/jds.2018-14830;HT-MF
48. Schuck, P. , Piot, M. , Méjean, S. , Fauquant, J. , Brulé, G. , Maubois, J. L. ;"Déshydratation des laits enrichis en caséine micellaire par microfiltration ; comparaison des propriétés des poudres obtenues avec celles d’une poudre de lait ultra-propre";;Journal of Membrane Science;117;;P 261-270;;1996;https://doi.org/10.1016/0376-7388(96)00076-2;HT-MF
49. Schuck, P. , Piot, M. , Méjean, S. ,Le Graet, Y. , Fauquant, J. , Brulé, G. , Maubois, J. L. ;Déshydratation par atomisation de phosphocaséinate natif obtenu par microfiltration sur membrane;;Journal of Dairy Research;58;1;P 39-50.;;1991;http://doi.org/10.1017/S0022029900033495;HT-MF
50. Thomann, S., Schenkel, P., Hinrichs, J.;The impact of homogenization and microfiltration on rennet-induced gel formation;;Journal of Dairy Science;95;10;P 5510-5526.;;2012;http://doi.org/10.3168/jds.2011-4875;HT-MF-DF
51. Tremblay-Marchand, D., Doyen, A., Britten, A., Pouliot, Y.;A process efficiency assessment of serum protein removal from milk using ceramic graded permeability microfiltration membrane;;Journal of Dairy Science;97;5;P 2619-2632;;2014;http://dx.doi.org/10.3168/jds.2013-7635;HT-MF-DF
52. Vadi, P. K. , Rizvi, S. S. H.;Experimental evaluation of a uniform transmembrane pressure crossflow microfiltration unit for the concentration of micellar casein from skim milk;;Journal of Texture Studies;39;4;P326-344.;;2008;http://doi.org/10.1111/j.1745-4603.2008.00146.x;HT-MF
53. Zulewska, J. , Barbano, M.;Influence of casein on flux and passage of serum proteins during microfiltration using polymeric spiral-wound membranes at 50°C;;Thèse de doctorat en Biochimie, biologie cellulaire et moléculaire - Université des Sciences et Techniques du Languedoc;;;;;1987;;HT-MF
54. Zulewska, J. , Barbano, M.;The effect of linear velocity and flux on performance of ceramic graded permeability membranes when processing skim milk at 50°C;;Journal of Dairy Science;87;11;P 3601-3613.;;2004;http://doi.org/10.3168/jds.S0022-0302(04)73498-0;HT-MF
55. Zulewska, J. , Kowalik, J. , Lobacz, A. , Dec, B.;Short communication : Calcium partitioning during microfiltration of milk and its influence on rennet coagulation time;;Lait;79;3;P 347-354.;;1999;;HT-MF
56. Zulewska, J., Newbold, M., Barbano, D. M.;Efficiency of serum protein removal from skim milk with ceramic and polymeric membranes at 50°C;;Journal of Dairy Science;88;12;P 4183-4194;;2005;http://doi.org/10.3168/jds.S0022-0302(05)73104-0;HT-MF-DF
